# The role and regulatory mechanism of m^6^A methylation in the nervous system

**DOI:** 10.3389/fgene.2022.962774

**Published:** 2022-09-01

**Authors:** Lingling Jiang, Xiaoheng Li, Shasha Wang, Zengqiang Yuan, Jinbo Cheng

**Affiliations:** ^1^ Hengyang Medical College, University of South China, Hengyang, China; ^2^ The Brain Science Center, Beijing Institute of Basic Medical Sciences, Beijing, China; ^3^ Center on Translational Neuroscience, College of Life & Environmental Science, Minzu University of China, Beijing, China

**Keywords:** m^6^A methylation, nervous system, development, neurological disorders, mechanism

## Abstract

N6-methyladenosine (m^6^A) modification regulates RNA translation, splicing, transport, localization, and stability at the post-transcriptional level. The m^6^A modification has been reported to have a wide range of effects on the nervous system, including neurogenesis, cerebellar development, learning, cognition, and memory, as well as the occurrence and development of neurological disorders. In this review, we aim to summarize the findings on the role and regulatory mechanism of m^6^A modification in the nervous system, to reveal the molecular mechanisms of neurodevelopmental processes, and to promote targeted therapy for nervous system-related diseases.

## Introduction

Epigenetic modification is the regulation of gene expression through a series of complex modifications without changing the gene sequence, and mainly includes DNA modification, RNA modification, histone modification, and chromatin remodeling (Jones et al., 2016). Among these, RNA modification is the most common. Different types of RNA modifications have been identified, including N^6^-methyladenosine (m^6^A) (Dominissini et al., 2012), 5-methylcytidine (m^5^c) (Edelheit et al., 2013) and N^1^-methyladenosine modification (m^1^A) (Dominissini et al., 2016; Li and Liu, 2016). Among these, m^6^A is the most abundant and reversible chemical modification of eukaryotic mRNA (Liu and Pan, 2016). An increasing number of studies have shown that m^6^A plays an important role in RNA stability, mRNA translation, alternative splicing, and subcellular RNA localization (Zhao et al., 2017; Yang et al., 2018; Shi et al., 2019).

The m^6^A modification and dynamic changes are regulated by the cooperation of m^6^A methyltransferase (writer), m^6^A demethylase (eraser), and m^6^A methylation binding protein (reader). Multiple studies have shown that m^6^A modification is highly expressed in the brain tissue and plays a critical role in the functions of the nervous system. Therefore, in this review, we mainly focus on the function and regulatory mechanisms of m^6^A methylation in the nervous system.

## m^6^A modification-related proteins

**FIGURE 1:**
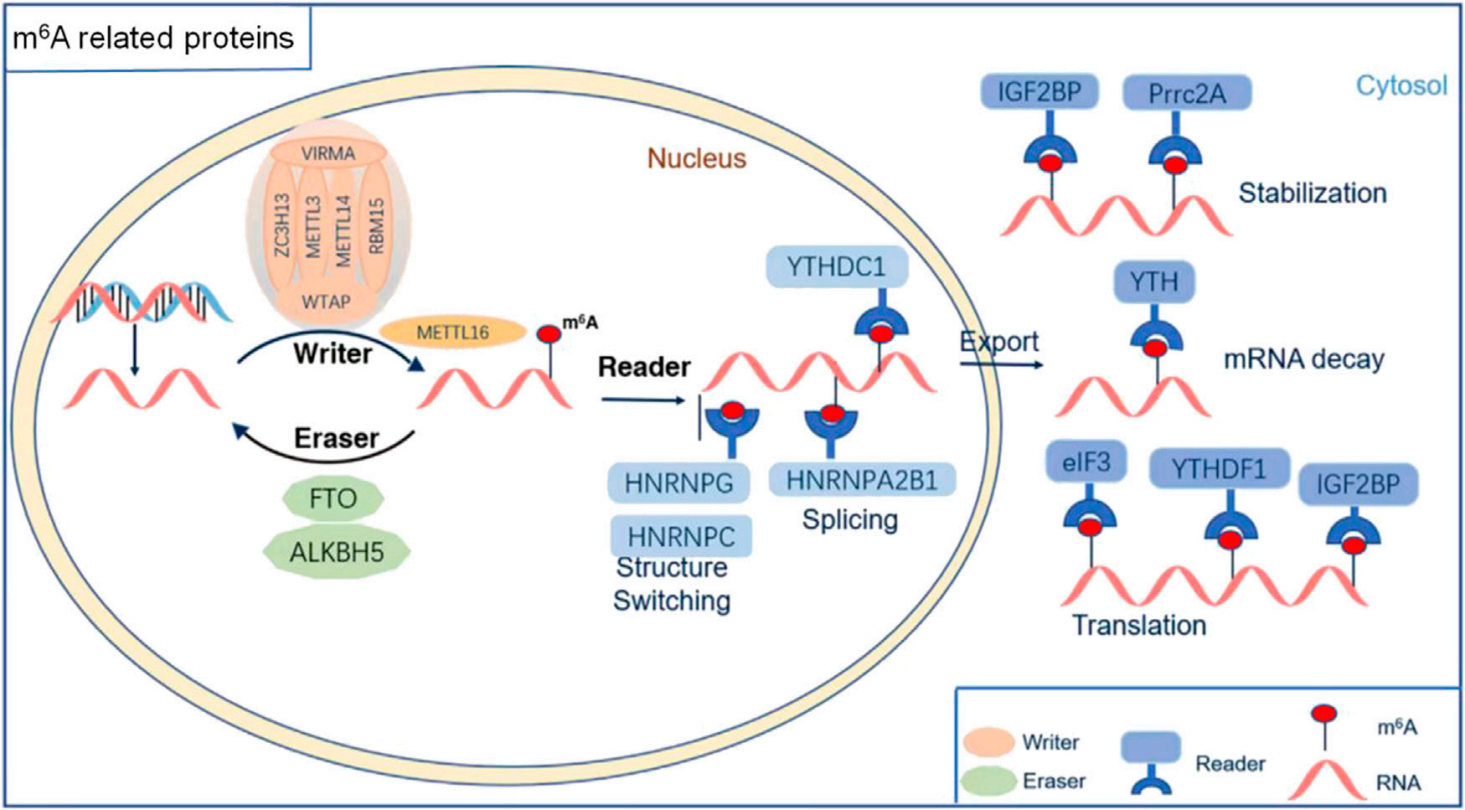
The molecular mechanism of RNA m^6^A modification. m^6^A methylation was catalyzed by the writer complex, including METTL3, METTL14, METTL16, WTAP, KIAA1429, RBM15/15B, and ZC3H13. The m^6^A modification was erased by demethylases, including FTO and ALKBH5. The m^6^A-modified RNA reader proteins included YTHDF1/2/3, YTHDC1/2, IGF2BP1/2/3, HNRNPC/G, HNRNPA2B1, Prrc2A, and eIF3.

**FIGURE 2:**
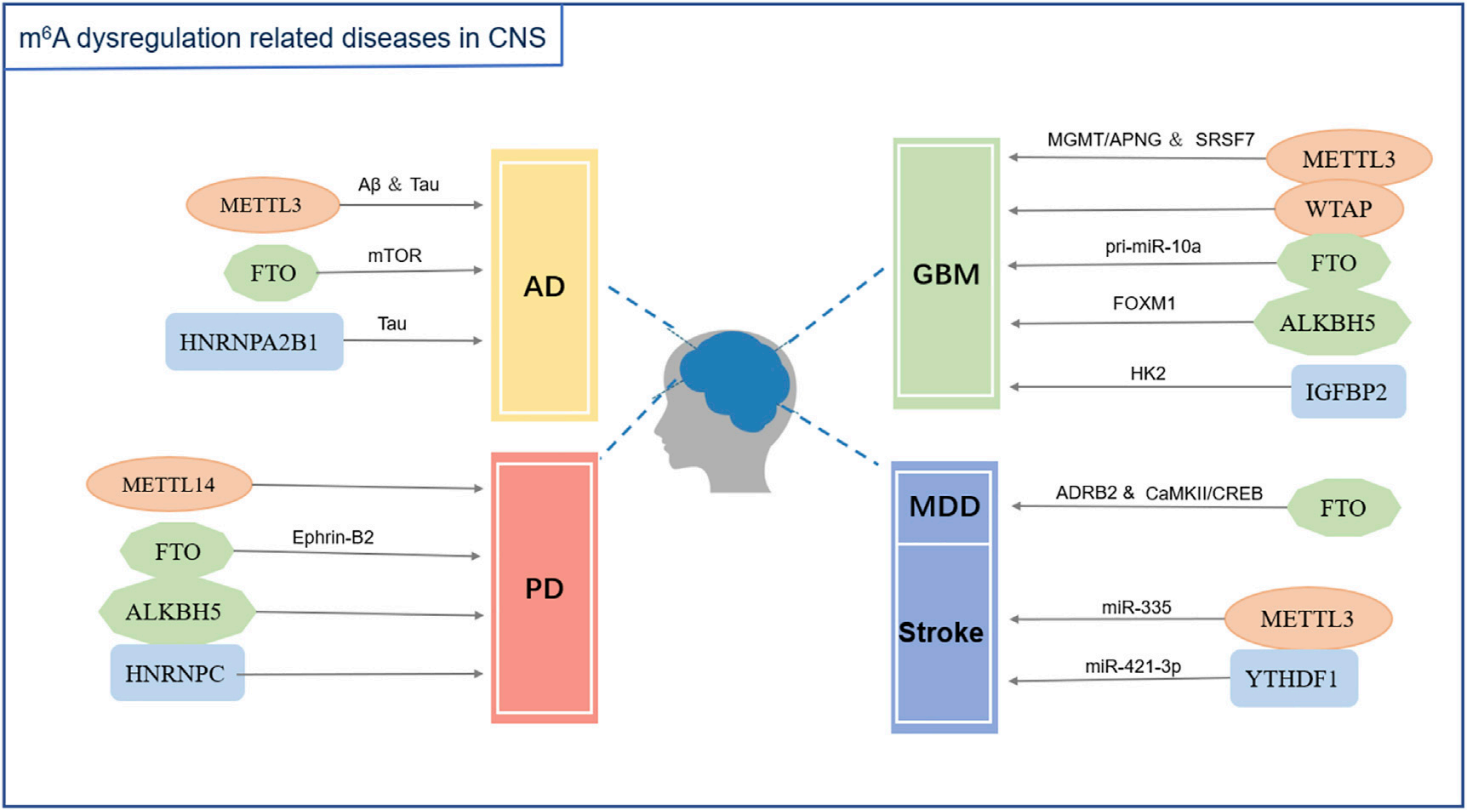
m^6^A modification dysfunction related diseases in the central nervous system. Abbreviations: AD (Alzheimer’s disease), PD (Parkinson’s disease), GBM (Glioblastoma), MDD (Major depressive disorder), Aβ (amyloid-β), Tau (Tau protein), mTOR (Mammalian target of rapamycin signaling pathway), Ephrin-B2 (erythropoietin-producing hepatocyte receptor-interacting-B2), MGMT (O^6^-methylguanine-DNA methyltransferase), APNG (alkylpurine-DNA-N-glycosylase), SRSF7 (Serine/arginine-rich splicing factor 7), pri-miR-10a (primary microRNA-10a), FOXM1 (transcription factor FOXM1), HK2 (Hexokinase 2), ADRB2 (Adrenoceptor beta 2), CaMKII (Calcium-calmodulin-dependent protein kinase II), CREB (cAMP response element-binding protein), miR-335 (microRNA-335), miR-421-3P (microRNA-421-3p).

A variety of RNA methyltransferases and demethylases as well as various m^6^A-binding proteins are present in the cells. Different types of RNAs undergo dynamic changes in methylation and demethylation, which, in turn, participate in the regulation of various physiological processes. With the development of molecular biology detection technology, an increasing number of enzymes and regulatory proteins related to m^6^A modifications have been discovered. Moreover, their roles in the development, homeostasis, and pathological state of the nervous system have been revealed (Figures 1, 2; Table 1).

**TABLE 1:** Functions of m^6^A modification-related proteins.

Type	Genetic name	Function	References
m^**6**^A writer	METTL3	Identify the conservative base sequence of m^6^A, catalytic methylation modification	Geula et al. (2015)
	METTL14	Assist METTL3 to catalytic methylation modifications	Wang et al. (2016)
	WTAP	Promote METTL3 and METTL14 heterogeneous dilate	Agarwala et al. (2012)
	VIRMA	Special modification of the 3′UTR region	Yue et al. (2018)
	RBM15	Combined with the m^6^A complex and recruit it to a special RNA site	Patil et al. (2016)
	ZC3H13	Connect to WTAP to mRNA binding factor Nito	Wen et al. (2018)
	METTL16	Catalytic methylation modification	Pendleton et al. (2017)
m^**6**^A eraser	FTO	Delete methylation modification	Jia et al. (2011)
	ALKBH5	Delete methylation modification	Zheng et al. (2013)
m^**6**^A reader	YTHDC1	Recruit splicing factors to splicing target mRNAs, promoting mRNA output degradation and specific transcription	(Xiao et al., 2016; Roundtree et al., 2017; Shima et al., 2017)
	YTHDC2	Adjust the stability and translation of mRNA	Hsu et al. (2017)
	YTHDF1	Recruit translation start factor to promote the translation of mRNA	Wang et al. (2015)
	YTHDF2	Combined with m^6^A modifiers to recruit CCR4-NOT deadenylase complex acceleration destination mRNA degradation	Du et al. (2016)
	YTHDF3	Promote mRNA degradation or medium translation	(Li et al., 2017a; Shi et al., 2017)
	HNRNPA2B1	Promote primary microRNA processing	Wu et al. (2018)
	HNRNPC/G	Mediate mRNA splicing	(Liu et al., 2015; Wang et al., 2015)
	IGF2BP1/2/3	Improve the stability of mRNA	Huang et al. (2018)
	eIF3	Combined with m^6^A to start translation	Meyer et al. (2015)
	FMRP	Adjust the stability, translation and nuclear output of mRNA	Santoro et al. (2012)
	Prrc2A	Improve the stability of mRNA	Wu et al. (2019)

### m^6^A methyltransferase

m^6^A methylation in mRNA is generally completed by two methyltransferases: METTL3/METTL14 and Wilms’ tumor 1-associating protein (WTAP) (Liu et al., 2014; Ping et al., 2014). METTL14 can assist METTL3 with catalytic methylation modifications (Wang et al., 2016) and WTAP promotes the formation of the WTAP/METTL3/METTL14 complex (Agarwala et al., 2012). Other constituent proteins of the complex include virus-like m^6^A methyltransferase-related protein (KIAA1429/VIRMA) (Yue et al., 2018), zinc finger CCCH domain-containing protein 13 (ZC3H13) (Wen et al., 2018), RNA-binding motif protein 15 and its paralog RBM15B (RBM15/15B) (Patil et al., 2016). Another methyltransferase is METTL16 (Pendleton et al., 2017).

METTL3 is the first discovered m^6^A methyltransferase component and acts as a core component of the methyltransferase complex. METTL3 is an S-adenosylmethionine-dependent methyltransferase that specifically recognizes the conserved m^6^A motif RRACH (R = A or G; H = A, C, or U) (Narayan and Rottman, 1988; Harper et al., 1990). METTL14 is a homologous protein of METTL3, which can form a dimer with METTL3 in a ratio of 1:1 (Liu et al., 2014). Knockout of METTL3 depletes m^6^A modifications in mRNA, which have been shown to cause early embryonic lethality in mice (Geula et al., 2015). Similar to METTL3, deletion of METTL14 leads to an equivalent reduction in m^6^A modifications in mRNA, and exhibits resistance to differentiation as seen in METTL3 knockout ESCs (Geula et al., 2015), suggesting a critical role of m^6^A modification *in vivo* (Liu et al., 2014). The depletion of WTAP significantly reduces the abundance of m^6^A in cellular mRNA, and its absence strongly reduces the binding ability of METTL3 to other components of the methyltransferase complex, suggesting that WTAP might target RNA by recruiting m^6^A methyltransferase complex (Ping et al., 2014).

An increasing number of proteins that interact with the core methyltransferase complex have been identified. VIRMA, a new subunit of the m^6^A methyltransferase complex, was found to regulate m^6^A methylation in the region near the 3′UTR and stop codon and is thus associated with alternative polyadenylation (Yue et al., 2018). RNA binding motif protein 15 (RBM15) and its paralog RBM15B can bind to the m^6^A methyltransferase complex and recruit it to specific sites in RNA. Knockdown of RBM15 and RBM15B impairs gene silencing mediated by X-inactive specific transcript (XIST, a long non-coding RNA that mediates the silencing of genes on the X chromosome) (Patil et al., 2016). In addition, zinc finger CCCH domain-containing protein 13 (ZC3H13) has been reported to be essential for methyltransferase complex localization. Knockdown of ZC3H13 in mouse embryonic stem cells was shown to significantly decrease global m^6^A mRNA levels (Wen et al., 2018). METTL16 is another indispensable methyltransferase, which regulates U6 small nuclear RNA (snRNA) methyltransferase and adenosyl transferase 2A levels (Pendleton et al., 2017; Doxtader et al., 2018). Unlike the METTL3 preferred m^6^A modification motif RRACH, METTL16 uses a structured RNA carrying a specific nonameric sequence of UAC**A**GAGAA (Mendel et al., 2018).

### m^6^A demethylase enzyme

m^6^A modification is a dynamic, reversible process. Demethylases, including fat mass and obesity-associated protein (FTO) and alkylation repair homologue 5 (ALKBH5), both belong to the non-heme Fe (II)- and α-KG-dependent dioxygenase ALKB dioxygenase family, and are responsible for eliminating m^6^A methylation modification in RNA (Jia et al., 2011; Zheng et al., 2013).

FTO is the first discovered m^6^A demethylase. It was originally recognized as a demethylase that mediates demethylation of 3-methylthymine in single-stranded DNA (Gerken et al., 2007) and 3-methyluracil in single-stranded RNA (Jia et al., 2008). Overexpression of FTO significantly decreases m^6^A content, whereas FTO knockdown increases m^6^A mRNA levels (Jia et al., 2011).

ALKBH5 affects mRNA export and RNA metabolism, as well as the assembly of mRNA processing factors in nuclear speckles. ALKBH5 deficiency increases m^6^A mRNA levels and impairs fertility in mice (Zheng et al., 2013). In addition to FTO and ALKBH5, additional m^6^A demethylases remain to be discovered.

### m^6^A binding proteins

The functioning of m^6^A methylation requires the recognition and binding of specific RNA-binding proteins. At present, the known m^6^A-binding proteins in mammals include YTH N6-Methyladenosine RNA Binding Protein 1 (YTHDF1), YTHDF2, YTHDF3, YTH domain containing 1 (YTHDC1), and YTHDC2 in the cytoplasm, heterogeneous nuclear ribonucleoprotein A2/B1 (HNRNPA2B1), Heterogeneous Nuclear Ribonucleoprotein C (HNRNPC), and Heterogeneous Nuclear Ribonucleoprotein G (HNRNPG) in the nucleus.

YTHDF1 is a cytoplasmic m^6^A-binding protein that does not have a critical effect on the stability of mRNA, but mainly promotes the translation of target mRNA by recruiting translation initiation factors (Wang et al., 2015). YTHDF2 is an m^6^A-binding protein that mediates mRNA translation, thereby affecting RNA half-life, accelerating RNA degradation, and mRNA stability (Wang et al., 2014). Upon binding to the m^6^A modification site, YTHDF2 accelerates target mRNA degradation by recruiting the CCR4-NOT deadenylase complex (Du et al., 2016). YTHDF3 either interacts with YTHDF2 to promote mRNA degradation or interacts with YTHDF1 to promote translation (Li et al., 2017a; Shi et al., 2017). Moreover, YTHDC2 is a cytoplasmic m^6^A-binding protein that regulates mRNA stability and translation and regulates spermatogenesis (Hsu et al., 2017; Wojtas et al., 2017). YTHDC1 is a nuclear m^6^A-binding protein that promotes exon inclusion in targeted mRNAs by recruiting the pre-mRNA splicing factor Ser/Arg-rich splicing factor 3 (SRSF3) (Xiao et al., 2016). YTHDC1 knockdown results in the accumulation of transcripts in the nucleus and subsequent depletion in the cytoplasm, suggesting a critical role in the metabolism of mRNAs (Roundtree et al., 2017). Interestingly, it has been reported that cytoplasmic YTHDC1 is involved in the mRNA maturation process, involving MAT2A (Shima et al., 2017).

Moreover, HNRNPA2B1, an m^6^A reader in the nucleus, has been shown to recognize and bind m^6^A-modified RNA, and regulate the maturation, transport, and metabolism of mRNA and the maturation of gene regulation of long noncoding RNAs (Alarcón et al., 2015; Wu et al., 2018), suggesting its role as a critical reader in m^6^A marking and its subsequent effects. In addition, insulin-like growth factor binding proteins (IGF2BP1/2/3), another set of nuclear m^6^A readers, have been shown to enhance the stability and translation of mRNA (Huang et al., 2018).

HNRNPC and HNRNPG are the nuclear RNA-binding proteins responsible for pre-mRNA processing. The binding ability of HNRNPC and HNRNPG is regulated in an m^6^A-switch dependent manner, illustrating a new mechanism of RNA-modification-coded cellular biology (Liu et al., 2015; Liu et al., 2017). In addition to the above, there are other m^6^A-binding proteins that can recognize and bind m^6^A modified sites, such as the translation initiation factor eukaryotic initiation factor 3 (eIF3), which selectively binds to m^6^A-modified RNAs. Upon binding with eIF3, 5′UTR m^6^A residues stimulate translation initiation (Meyer et al., 2015). Furthermore, proline rich coiled-coil 2A (Prrc2A), a novel m^6^A specific binding protein, has been shown to bind with m^6^A modified mRNA and regulate myelination of oligodendrocytes (Wu et al., 2019).

The RNA-binding protein, fragility X mental retardation protein (FMRP), is encoded by *FMR1*. FMRP is critical in metabotropic glutamate receptor-dependent long-term depression and synaptic plasticity. The absence of FMRP causes excessive and persistent protein synthesis in postsynaptic dendrites and dysregulated synaptic functions (Santoro et al., 2012).

## The role of m^6^A in the development of the nervous system

The process of nervous system development is highly coordinated by different cell types, and has the potential for self-renewal and the ability to produce many types of differentiated cells. The gene expression of neural cells is regulated by many factors, including various epigenetic modifications, such as m^6^A modification. As reported, a high abundance of m^6^A modifications exist in the nervous system (Livneh et al., 2020). In the brain, the level of m^6^A increases from embryonic stage to adulthood (Jiang et al., 2021), and nearly half of the mRNAs stably expressing cerebral cortex genes in adult mice have m^6^A methylation modification (Chang et al., 2017), suggesting that m^6^A methylation modification plays an important role in the nervous system.

### m^6^A methylation in neurogenesis

Neurogenesis is the process of producing neurons from different types of neural progenitor cells (NPCs, including neural stem cells), most of which occurs during embryonic development (Florio and Huttner, 2014). Neurogenesis is regulated by multiple factors, among which m^6^A methylation is particularly important for the regulation of neurogenesis, and an imbalance in m^6^A methylation levels can cause neurogenesis abnormalities.

The m^6^A methyltransferase, METTL3, affects neurogenesis and neuronal development, and its deletion significantly reduces m^6^A levels, affects the proliferation and cell cycle process of adult neural stem cells (aNSCs), and inhibits the morphological maturation of newborn neurons. Meanwhile, overexpression of the histone methyltransferase Ezh2 was shown to rescue neurogenesis and neuronal development defects caused by METTL3 deletion (Chen et al., 2019a). METTL14, one of the core molecules of the m^6^A methyltransferase complex, mediates m^6^A modification to participate in neurogenesis by modulating cell cycle progression of cortical neural progenitors. Loss of METTL14 results in the nuclear accumulation of mRNA associated with neural differentiation and delayed differentiation of mouse neural progenitor cells (Yoon et al., 2017; Edens et al., 2019). FTO is widely expressed and highly enriched in neuronal and adult neural stem cells. It is closely related to the proliferation and differentiation of aNSCs, and its deficiency can reduce proliferation and neuronal differentiation of aNSCs (Li et al., 2017b). Another study has reported that FTO dynamically influences neurogenesis by modulating the Pdgfra/Socs5-Stat3 pathway (Cao et al., 2020).

Conditional depletion of YTHDF2 leads to embryonic lethality, suggesting that YTHDF2 plays a critical role in early embryonic development. YTHDF2-mediated m^6^A epitranscriptomics regulates cortical neurogenesis during embryonic neural development by regulating RNA degradation of m^6^A-tagged genes associated with neural development and differentiation (Li et al., 2018a). Moreover, YTHDF2 can destabilize m^6^A-modified neural-specific RNA, thus inhibiting the differentiation of induced pluripotent stem cells (Heck et al., 2020). Furthermore, decline in Imp (IGF2BP) levels, a highly conserved RNA-binding protein, has been shown to limit the stability of myc mRNA and restrict neuroblast growth and division in *Drosophila* (Samuels et al., 2020). Another study reported that deletion of Fmr1 is associated with abnormalities in cortical development and dendritic spine formation, which leads to abnormal learning and behavior in fragile X syndrome (Castrén et al., 2005; Saffary and Xie, 2011; La Fata et al., 2014). Loss of Fmr1 results in nuclear retention of m^6^A-modified FMRP target mRNAs that regulate neural differentiation, ultimately resulting in defective neuronal function (Edens et al., 2019).

In addition to being essential for neuronal development, m^6^A also plays an important role in the development of glial cells in mammals. Oligodendrocytes are glial cells in the central nervous system that are responsible for myelination of axons (Nave and Werner, 2014; Simons and Nave, 2015). METTL14-mediated m^6^A methylation is critical for regulation of oligodendrocyte development and myelination of the central nervous system. Conditional inactivation of METTL14 leads to a decrease in the number of oligodendrocytes and CNS hypomyelination. Moreover, dynamic changes in m^6^A transcription affect the development of oligodendrocyte lineage (Xu et al., 2020a). In our study, we found that Prrc2a regulates the myelination of oligodendrocytes. Conditional knockout of Prrc2a in mice leads to pathological features, such as inadequate myelination, shortened lifespan, and impaired motor and cognitive function. Mechanistically, Prrc2a increases the stability of oligo2 mRNA in an m^6^A-dependent manner, thereby regulating the proliferation and fate of oligodendrocytes (Wu et al., 2019).

### m^6^A methylation in the formation and function of neuronal system

m^6^A methylation plays an important role in axonal and synaptic growth, and dysregulation of m^6^A modification leads to abnormal synaptic function. For example, loss of METTL14 or YTHDF1 attenuates injury-induced protein translation in adult dorsal root ganglion neurons (DRGs) and decreases functional axon regeneration in the peripheral nervous system (Weng et al., 2018). FTO is highly expressed in neuronal axons, suggesting that it may play an important role in neuronal axon development and function (Li et al., 2017b). FTO knockdown in axons increases m^6^A levels, decreases local translation and expression of growth-associated protein 43 mRNA, and markedly inhibits axonal growth (Yu et al., 2018).

The YTHDF family of m^6^A methylation-binding proteins also limit the growth and development of axons by regulating the translation of related transcripts (Worpenberg et al., 2021). YTHDF1 is closely associated with axonal growth, development, and function. It physiologically regulates the translation of Robo3.1, which controls the guidance of pre-crossing axons in the embryonic spinal cord (Zhuang et al., 2019). In addition, YTHDF1/2 is closely related to axonal growth in granulosa cells. Knockdown of YTHDF1/2 promotes axonal growth in granule cells by regulating the local translation of Wnt5a signaling components (Yu et al., 2021). Knockdown of YTHDF1 in hippocampal neurons results in synaptic dysfunction, including immature dendritic spine morphology and inhibition of excitatory synaptic transmission, accompanied by a reduction in postsynaptic density 95 (PSD-95) and decreased surface expression of GluA1 of AMPA receptors (Merkurjev et al., 2018).

### m^6^A in learning cognitive function

METTL3-mediated m^6^A methylation is involved in the formation of long-term memory in the hippocampus. Depletion of METTL3 in the mouse hippocampus reduces memory consolidation. Moreover, adequate training or restoration of METTL3 could rescue learning memory defect (Zhang et al., 2018). In addition, m^6^A methylation modification in glutamatergic neurons has been reported to be important for context fear memory generalization (Chang et al., 2022). Deletion of METTL3 in hippocampal glutamatergic neurons results in more freezing behavior, suggesting a lower discrimination index.

Another indispensable component of the m^6^A methyltransferase complex is METTL14, which has been shown to influence learning in mice. It was found that METTL14 was essential for striatal function and learning-related transcriptional regulation, and conditional neuron-specific striatal deletion of METTL14 reduced m^6^A methylation levels and impaired striatum-mediated behavior and learning abilities (Koranda et al., 2018). Additionally, FTO plays an important role in the formation of memory in the cerebral cortex and hippocampus. FTO deficiency could reduce the proliferation of aNSCs and neuronal differentiation, resulting in impaired neurogenesis as well as learning and memory defects (Li et al., 2017b). The level of m^6^A modification in the prefrontal cortex of mice increases with behavioral experience, and targeted knockdown of FTO in the prefrontal cortex significantly enhances the consolidation of cued fear memory (Widagdo et al., 2016). In addition, m^6^A-binding proteins play a key role in learning and memory processes. YTHDF1 facilitates learning and memory, and deletion of YTHDF1 impairs synaptic transmission and long-term potentiation. YTHDF1 facilitates the translation of m^6^A-methylated neuronal mRNAs in response to neuronal stimulation, which is necessary for learning and memory. (Shi et al., 2018). Moreover, the YTHDF family mediated m^6^A modification was also shown to affect learning and memory formation in *Drosophila* (Kan et al., 2021).

### m^6^A modification in cerebellar development

m^6^A modification is also indispensable in the development of the cerebellum. The m^6^A-related genes METTL3, METTL14, WTAP, FTO, and ALKBH5 in mice showed a decreasing trend with age during cerebellar development, with the highest level in P7, which decreased and stabilized after P14. Dynamic regulation of METTL3-mediated m^6^A methylation modification is associated with cerebellar development (Ma et al., 2018; Wang et al., 2018).

Conditional deletion of METTL3 using *Nestin-Cre* in the nervous system causes severe developmental defects in the brain. Mechanistically, deletion of METTL3 significantly increases the apoptosis of newborn cerebellar granulocytes (CGCs) and impairs the maturation of Purkinje cells (Wang et al., 2018). Consistently, knockdown of METTL3 by lentivirus was shown to result in a severe alteration in Purkinje cell numbers, laminal structure, and stunted dendrites and caused defects in mouse cerebellar development. In addition, ALKBH5 deletion affects RNA nuclear output, impairs RNA metabolism, and affects cerebellar development (Ma et al., 2018). Furthermore, WTAP has been found to be closely related with cerebellar development in mice, and WTAP deletion leads to Purkinje cell degeneration, cerebellar ataxia, and cerebellar atrophy (Yang et al., 2022). The m^6^A-binding proteins also play an important role in the development of the cerebellum, and specific knockout of YTHDF1 or YTHDF2 in granule cells promotes the growth of parallel fibers and the formation of cerebellar synapses, thereby improving motor coordination in mice (Yu et al., 2021). Together, these studies suggest that m^6^A plays a critical role in neurogenesis, cerebellar development, and cognitive function, which are indispensable for proper physiological functioning of the nervous system (Table 1).

## m^6^A methylation in neurological disorders

m^6^A methylation is widely present in mammalian transcripts that affect the basic metabolism of RNA and participate in the dynamic regulation of various biological processes. In recent years, multiple studies have shown that m^6^A methylation is closely related to nervous system diseases. The level of m^6^A methylation changes in the pathological state. Simultaneously, the dysregulation of m^6^A methylation modification also causes neurodevelopmental abnormalities and neurological diseases (Figure 2; Table 2).

**TABLE 2:** Functions of m^6^A modification-related proteins in neurological diseases.

Disease type	m^6^A regulator	Function	References
Alzheimer’s disease	METTL3	Involve in the pathogenesis of AD	Zhao et al. (2021)
	FTO	regulate the mTOR signaling pathway and affected the development of AD	Li et al. (2018b)
	HNRNPA2B1	Involved in the regulation of AD	Kolisnyk et al. (2016)
Parkinson’s Disease	METTL14	Affect the production of key enzymes required for dopamine synthesis	Teng et al. (2021)
	FTO	Maintain dopaminergic neuron function	(Hess et al., 2013; Qi et al., 2022)
	HNRNPC	regulate dopaminergic proliferation	Quan et al. (2021)
	ALKBH5	Relate to the risk of PD	Qiu et al. (2020)
Major depressive disorder	FTO	Target ADRB2 induces depression-like behaviors	Liu et al. (2021a)
	FTO	Target the CaMKII/CREB signaling pathway modulates hippocampal synaptic plasticity	Shen et al. (2021)
	FTO	Affect resistance to tricyclic antidepressants	Wu et al. (2021)
	FTO	regulate the function of astrocytes	Huang et al. (2020b)
Stroke	METTL3	Involve in the formation of early stress granule cells	Si et al. (2020)
	YTHDF1	Involve in inflammatory response in stroke model mice	Zheng et al. (2020)
	YTHDC1	Influence the degradation of PTEN mRNA	Zhang et al. (2020)
Glioblastoma	METTL3	Influence self-renewal capacity of GBM cells	Li et al. (2019)
	METTL3	Influence the development process of GBM	Cui et al. (2017)
	METTL3	Influence tumor growth and development	Visvanathan et al. (2018)
	METTL3	Maintain specific gene expression in GSCs and regulate oncogenic-related signaling pathways	Visvanathan et al. (2019)
	METTL3	Regulates DNA repair factors and affects cellular drug resistance	Shi et al. (2021a)
	WTAP	Involve in carcinogenesis	Jin et al. (2012)
	FTO	Affect GBM cell proliferation, migration and invasion	Zhang et al. (2022)
	ALKBH5	Influence the GSCs self-renewal and tumorigenesis	Zhang et al. (2017)
	ALKBH5	Influence the radioresistance and invasive ability of GSCs	Kowalski-Chauvel et al. (2020)
	YTHDF2	Influence the development process of GBM	Dixit et al. (2021)
	YTHDF2	Influence the GBM cell proliferation, invasion, and tumorigenesis	Fang et al. (2021)
	YTHDF2	Involve in UBXN1 mRNA degradation	Chai et al. (2021)
	IGFBP1/2/3	Affects the formation of GBM microenvironment, regulates carcinogenic factors, affects GBM aerobic glycolysis, and affects GBM prognosis	(Liu et al., 2021b; Sun et al., 2021; Xiong et al., 2022)

### m^6^A methylation in Alzheimer’s disease (AD)

AD is a neurodegenerative disease characterized by progressive cognitive dysfunction and impaired learning and memory (Lane et al., 2018). As mentioned above, m^6^A plays a critical role in learning functions, such as cognitive and memory formation and consolidation (Weng et al., 2018; Zhang et al., 2018). It has been reported that m^6^A methylation also occurs during the occurrence and development of AD. The m^6^A modification levels were shown to be significantly reduced in pyramidal neurons in AD patients, whereas the m^6^A level in glial cells was significantly increased (Zhao et al., 2021). The abundance of m^6^A modification was shown to be significantly lower in 5×FAD mouse brain than in control mice (Shafik et al., 2021). However, it was reported that the levels of m^6^A methylation in the cortex and hippocampus of APP/PS1 transgenic mice were higher than those in the control group (Han et al., 2020). Differences in mouse models and brain tissue might contribute to these contradictory results. In addition, m^6^A methyltransferases METTL3, METTL14, and WTAP; demethylase FTO; and m^6^A binding protein YTHDF1 were found to be significantly reduced in the frontal cortex tissues of patients with AD. Notably, the expression of METTL3 was significantly downregulated in the brains of patients with mild cognitive impairment (Zhao et al., 2021). In addition, METTL3 accumulates in amyloid-β (Aβ) plaques and intracellular tau nerve fiber tangles, and its accumulation levels are positively correlated with tau protein levels (Huang et al., 2020a). Abnormal expression and distribution of m^6^A modification-related proteins in the hippocampus of patients with AD suggests that m^6^A modification is associated with the pathogenesis of AD. In an AD mouse model, METTL3 knockdown in the hippocampus resulted in cognitive dysfunction, synaptic loss, neuronal death, increased oxidative stress, and abnormal cell cycle events. Overexpression of METTL3 in neurons salvaged Aβ-induced synaptic damage and cognitive impairment (Zhao et al., 2021). METTL3 levels were elevated in the cortical and hippocampal brain regions of AD mice, whereas FTO levels were decreased in the hippocampal brain region (Han et al., 2020). However, one study reported that FTO levels were upregulated in AD mouse models, and overexpression of FTO activated the mTOR signaling pathway to increase phosphorylated tau protein levels, which promoted the development of AD (Li et al., 2018b). Moreover, the m^6^A-binding protein HNRNPA2B1 was shown to participate in the pathological process of AD (Kolisnyk et al., 2016). Although the role of m^6^A methylation in AD is not yet exhaustive, the study of m^6^A modification provides a new perspective target for the treatment of AD.

### m^6^A methylation in Parkinson’s disease (PD)

PD is a common age-related neurodegenerative disease, but its pathogenesis has not been fully elucidated. The loss of dopaminergic neurons in the substantia nigra of the midbrain leads to a decrease in the content of dopamine in the striatum, resulting in a decrease in the activity of the substantia nigra–striatum dopamine transmitter system and relative hyperactivity of cholinergic neurons in the striatum (Schapira and Jenner, 2011). m^6^A methylation modifications are highly abundant in the brain and play a vital role in dopaminergic neuronal pathways in the midbrain. Specific deletion of FTO in dopaminergic neurons causes abnormal dopaminergic neuron function (Hess et al., 2013). FTO was shown to play a protective role in manganese-mediated PD models by antagonizing the downregulation of the axon-guided molecule ephrin-B2 (Qi et al., 2022). Specific knockout of methyltransferase METTL14 in the substantia nigra region was demonstrated to cause a decrease in the key enzyme of dopamine synthesis, tyrosine hydroxylase, which in turn caused motor dysfunction in mice (Teng et al., 2021). These studies suggest that an imbalance in the dynamic regulation of m^6^A is one of the causes of pathological changes in PD. Moreover, reduced levels of m^6^A modification in neurons were shown to increase the expression of N-methyl-d-aspartate receptor l, thereby increasing the inflow of calcium and oxidative stress, ultimately leading to apoptosis of dopaminergic neurons (Chen et al., 2019b). In addition, the m^6^A methylation recognition protein HNRNPC is significantly downregulated in PD, and it may be involved in the process of regulating PD by inhibiting the proliferation of dopaminergic neurons (Quan et al., 2021). In addition to animal experiments, m^6^A-associated mononucleotide polymorphisms in the rs1378602, rs4924839, and rs8071834 genes encoding the demethylase ALKBH5 in PD patients were found to be closely related to the risk of PD (Qiu et al., 2020). Although numerous studies have shown an important relationship between m6A modification and PD, the characteristics of m^6^A modification and the mechanisms that regulate the development of PD require further exploration.

### m^6^A methylation in depression

Major depressive disorder (MDD) is a common, chronic, and recurrent psychiatric disorder. Epigenetic RNA modifications are also involved in the pathogenesis of MDD. The RNA demethylase ALKBH5 was found to be closely related to MDD, suggesting that m^6^A methylation plays an important role in MDD (Du et al., 2015; Barbon and Magri, 2020). FTO expression has been shown to be downregulated in the hippocampus of MDD patients and mouse models of depression, and FTO in mouse hippocampal tissue was shown to induce depressive-like behavior in mice by targeting ADRB2 (Liu et al., 2021a). Similarly, FTO was demonstrated to target the CaMKII/CREB signaling pathway to regulate hippocampal synaptic plasticity, and activation of FTO in the hippocampus alleviated chronic restraint stress-induced depression-like behaviors in mice (Shen et al., 2021). In addition, the absence of FTO in the ventral covered area (VTA) of the midbrain was shown to increase the susceptibility of mice to stress and resistance to tricyclic antidepressants. Consistently, overexpression of FTO in the VTA significantly alleviated depression-like behavior in mice (Wu et al., 2021). Moreover, FTO deficiency has been shown to reduce anxiety- and depression-like behaviors (Sun et al., 2019). These contradictory results may be due to differences in the role of FTO in different brain regions. In addition, the cyclic RNA STAG1 (circSTAG1) was shown to alleviate astrocyte dysfunction by capturing ALKBH5, which in turn played an antidepressant role (Huang et al., 2020b). Together, these studies demonstrate that m^6^A methylation is involved in the regulation of MDD and provide new ideas for the treatment of MDD.

### m^6^A methylation in stroke

Several studies have shown that m^6^A methylation is closely related to ischemic stroke (Xu et al., 2020b; Yi et al., 2021). m^6^A methylation has been shown to be involved in post-stroke pathophysiological processes, such as inflammation, apoptosis, and transcriptional regulation, but the specific regulatory mechanisms need to be explored further (Chokkalla et al., 2019). The m^6^A methyltransferase METTL3 was shown to be associated with the formation of stress granules (SGs) in early acute ischemic stroke and reducing the apoptosis of injured neurons and cells by increasing the maturation of miR-335 through m^6^A methylation (Si et al., 2020). The m^6^A methylation-binding protein YTHDF1 was demonstrated to play an important role in cerebral ischemia-reperfusion injury. After cerebral ischemia reperfusion injury, the expression of miR-421-3p was significantly reduced, which inhibited the translation of p65 by targeting YTHDF1, thereby inhibiting the inflammatory response (Zheng et al., 2020). Another study found that knockdown of YTHDC1 aggravated ischemic brain injury, whereas overexpression of YTHDC1 increased phosphorylation of Akt by promoting the degradation of *PTEN* mRNA, thereby saving neurons after ischemia and protecting rats from cerebral ischemic damage (Zhang et al., 2020).

### m^6^A methylation in glioblastoma

Studies have shown that m^6^A modifications are widely involved in the occurrence, progression, and immune regulation of various cancers (Deng et al., 2018; Geng et al., 2020; Jiang et al., 2021; Uddin et al., 2021). Glioblastoma (GMB) is the most common primary malignant glioma in the central nervous system (Omuro and DeAngelis, 2013; Lapointe et al., 2018). m^6^A not only affects the clinical prognosis of GBM (Du et al., 2020) but is also closely related to GBM mesenchymal conversion and angiogenesis (Tao et al., 2022). In addition, m^6^A regulatory proteins are also associated with immune infiltration in GBM (Pan et al., 2021).

Several biological information analyses have shown that m^6^A regulatory proteins affect the prognosis and survival rate of GBM patients (Cai et al., 2021; Wang et al., 2021). METTL3-mediated m^6^A methylation promotes proliferation and self-renewal of GBM cells (Li et al., 2019). METTL3 knockout promotes the growth and self-renewal of human glioblastoma stem cells (GSCs) and promotes GBM development (Cui et al., 2017). In contrast, it was reported that METTL3 knockout in mouse GBM models inhibited tumor growth and development, and METTL3 knockdown in GSCs led to increased sensitivity of cells to radiation exposure (Visvanathan et al., 2018). In addition, METTL3 is indispensable in maintaining GSC-specific gene expression and positive regulation of carcinogenic-related signaling pathways (Visvanathan et al., 2019). These contradictory findings might be attributed to different GBM cell lines, differences in culture conditions, and genetic heterogeneity. In addition to influencing the occurrence and development of GBM, METTL3 is also related to resistance to chemotherapy drugs. METTL3 and m^6^A methylation levels have been shown to affect the sensitivity of temozolomide-resistant GBM cells. METTL3 regulates m^6^A methylation levels of DNA repair factors MGMT and APNG, thereby affecting glioma resistance to temozolomide (Shi et al., 2021a). Furthermore, SRSF7 plays an oncogenic role in various cancers. SRSF7 in GBM patients specifically targets and promotes the methylation of m^6^A sites in genes involved in cell proliferation and migration by recruiting a methyltransferase complex (Cun et al., 2021). Additionally, the horizontal expression of WTAP, a component of the m^6^A methyltransferase complex, is also strongly associated with poor prognosis in GBM (Xi et al., 2016). WTAP acts as a nucleoprotein associated with proliferation and apoptosis regulation and plays a carcinogenic role in gliomas (Jin et al., 2012). In addition to m^6^A methyltransferase, m^6^A demethylase and m^6^A recognition proteins are involved in regulating and influencing the occurrence and development of GBM. Reportedly, inhibition of FTO prevented neurosphere formation in patient-derived GSCs without inhibiting the growth of healthy neural stem cell-derived neurospheres (Huff et al., 2021). However, it was found that a decreased FTO level is closely related with poor prognosis. FTO regulates the maturation of miR-10a in an m^6^A-dependent manner and recruits the microRNA microprocessor complex protein DGCR8 through the recognition protein HNRNPA2B1, thereby promoting the proliferation, migration, and invasion of GBM cells (Zhang et al., 2022). ALKBH5 is highly expressed in GSCs. ALKBH5 in GBM stem-like cells enhances self-renewal and tumorigenesis by regulating FOXM1 (Zhang et al., 2017). In addition, ALKBH5 promotes radiation resistance and invasion of GSCs (Kowalski-Chauvel et al., 2020). In addition, the m^6^A methylation-binding protein YTHDF2 was found to affect tumor growth in mice through the YTHDF2-MYC-IGFBP3 pathway, providing a new target for the treatment of GBM (Dixit et al., 2021). YTHDF2 was shown to be associated with poor prognosis in glioma patients, and is required for GBM cell proliferation, invasion, and tumorigenesis (Fang et al., 2021). In addition, highly expressed YTHDF2 accelerates the degradation of UBXN1 mRNA by recognizing m^6^A modifications, thereby promoting the activation of NF-κB. UBXN1 overexpression attenuates YTHDF2 to promote malignant progression of glioma (Chai et al., 2021), suggesting that YTHDF2 might be involved in the occurrence and malignant progression of GBM. In addition, the m^6^A methylation binding proteins, including IGFBP1/2/3 are closely related to GBM. IGFBP1 was significantly positively correlated with most immune cells and matrix-related pathways, indicating that m^6^A methylation modification plays a role in the formation of the GBM microenvironment. IGF2BP2 interacts with the CASC9/IGF2BP2 complex and accelerates aerobic glycolysis in GBM by regulating the mRNA of HK2. It was reported that high expression of IGF2BP3 in patients with glioma significantly reduced survival time, which was also associated with poor prognosis, suggesting that IGF2BP3 might be used as a potential prognostic biomarker for gliomas (Liu et al., 2021b; Sun et al., 2021; Xiong et al., 2022). The important role of modified m^6^A-related proteins in GBM provides new insights into the prognosis and treatment of GBM.

### m^6^A methylation in other neuropathies

In addition to participating in the regulation of the above diseases, m^6^A is also closely related with other neurological diseases, such as amyotrophic lateral sclerosis, epilepsy, intellectual disability, schizophrenia, and traumatic brain injury (Batista, 2017; Jung and Goldman, 2018; Livneh et al., 2020). Mutations related to amyotrophic lateral sclerosis in the prion-like domain of hnRNPA could promote protein fibrosis in cells, suggesting that hnRNPA might be involved in the pathogenesis of atrophic lateral sclerosis (Kim et al., 2013). FTO is involved in regulating microRNAs and plays an important role in the physiology and pathology of the central nervous system (Rowles et al., 2012). METTL3-mediated m^6^A modification is involved in the dysregulation of NRIP1 expression in Down syndrome (Shi et al., 2021b). Deletion of the m^6^A methylated binding protein FMRP leads to fragile X syndrome (Santoro et al., 2012). In a traumatic brain injury rat model, it was shown that FTO plays an important role in maintaining neural function but has no effect on spatial learning and memory ability (Yu et al., 2020). In addition, m^6^A modification is involved in regulating drug toxicity-related nervous system damage. In a study in young mice, sevoflurane was shown to impair m^6^A-mediated mRNA translation and cause motor defects. YTHDF1 salvaged sevoflurane-induced suppression of protein synthesis and motor disorders in the young mice (Zhang et al., 2021). FTO has also been found to alleviate dopaminergic neurotransmission defects caused by exposure to arsenite and improve the progression of arsenic-related neurological disorders (Bai et al., 2018).

## Summary and outlook

In summary, as a very important epigenetic modification, m^6^A methylation regulates RNA translation, splicing, transport, localization, and stability at the post-transcriptional level. m^6^A methylation has a wide range of effects on nervous system development and function, including neurogenesis, cerebellar development, learning, cognition, and memory. It also plays an important role in the regulation of the occurrence and development of nervous system diseases. These studies provide new ideas and directions for revealing the molecular mechanisms of neurodevelopmental processes and promoting targeted therapies for neurological diseases.

Although research on m^6^A methylation has rapidly developed in recent years, there are still many unknowns. For example, the mechanism and function of some known m^6^A-related proteins in the nervous system have not been fully clarified, and there may be some undiscovered m^6^A methyltransferases, demethylases, and methylation recognition enzymes.
